# The risk of venous thromboembolism associated with peripherally inserted central catheters in ambulant cancer patients

**DOI:** 10.1186/s12959-017-0148-y

**Published:** 2017-09-19

**Authors:** Daniel Jones, Kurt Wismayer, George Bozas, June Palmer, Mandi Elliott, Anthony Maraveyas

**Affiliations:** 1 Queen’s Centre for Oncology and Haematology, Castle Hill Hospital, Hull and East Yorkshire Hospitals Trust, Hull, UK; 2 Hull York Medical School, York, UK; 3 Supportive care, Early Diagnosis and Advanced disease (SEDA) research group, Centre for Health and Population Sciences, University of Hull, Hertford Building, Hull, HU6 7RX UK

**Keywords:** Venous Thromboembolism, Peripherally Inserted Central Catheters, Deep Vein Thrombosis, Cancer, Risk Factors, Anticoagulation

## Abstract

**Background:**

Deep vein thrombosis (DVT) is a common complication of peripherally inserted central catheters (PICCs). PICCs are increasingly utilised in the management of cancer patients, a group which carries both additional risks for vascular thromboembolism as well as for complex morbidity. We analysed a cohort of cancer patients subjected to PICC insertion in a single cancer centre for the incidence of all-type vascular thromboembolism (VTE) and investigated relative risk factors.

**Methods:**

In this clinical audit, the records of patients referred for PICC insertion in our centre in the period between 1/1/2011 and 1/4/2014 were retrospectively reviewed. The primary outcomes investigated were a) PICC-related deep vein thrombosis (PRDVT) and b) distant VTE (lower limb DVT and pulmonary embolism). 4Fr single lumen PICCs were placed in all patients. The Kaplan Meier method was used to study time from PICC insertion to PRDVT/VTE. Survival curves were compared using the log rank method. Logistic and Cox regression analyses were used to assess local, distant and combined endpoints.

**Results:**

Four hundred ninety patients were included in the analysis of which 27 (5.5%) developed a PRDVT. Statistically significant risk factors for developing PRDVT in multivariate analysis included more than one attempt for insertion (OR 2.61, 95%CI: 1.12–6.05) and the use of fluoropyrimidine containing chemotherapy (OR 4.27, 95%CI 1.3–14.07). Twenty-six patients developed a distant VTE. Male gender was the only significant risk factor for distant VTE. When all-type VTE were considered together fluoropyrimidine containing chemotherapy (OR 4.54, 95% CI 1.63–12.61), male gender (OR 2.03, 95% CI 1.04–3.93) and white cell count (OR 1.12, 95% CI 1.00–1.26) were statistically significant as risk factors in this analysis.

**Conclusions:**

This is a large study of VTE following PICC insertion in cancer patients which also looks at the rate of distant VTE. The observed PRDVT incidence is comparable with available literature. Fluoropyrimidine containing chemotherapy and more than one attempt for PICC insertion were independent predictors of PICC-associated VTE whilst the former remained an independent predictor of all-type VTE. Anticoagulation did not prevent thrombotic events in this cohort.

## Background

The use of peripherally inserted central catheters (PICCs) is increasing, especially in the management of cancer patients. [1, 2]. PICCs can be fitted at the bedside by nurse-led teams, they are safer and more cost-effective than other central venous catheters (CVCs) [3–5]. Despite the benefits, PICC insertion has been shown to increase the risk of venous thromboembolism (VTE) particularly arm deep vein thrombosis (DVT) and pulmonary embolism (PE) [4, 6–8]. These events have been shown to occur commonly, to incur increased cost and to be associated with increased morbidity [9]. In cancer patients the risks may be higher [10]. A review addressing 16 studies, including 2169 cancer patients with CVC, found symptomatic DVT in 262 (12%) of patients [11]. This increased risk of VTE is important as these patients have increased morbidity and mortality, require prolonged antithrombotic treatment and may have medical treatment e.g. chemotherapy interrupted [3]. Very little work has been carried out looking at the incidence and risk factors for VTE in cancer patients following the insertion of PICCs. Published data has focused on all CVCs of which just 15% were PICCs [12]. Two cohort studies published in 2006 and 2012 found a PICC-related DVT (PRDVT) rate of 4.3 and 5.6% respectively with number of insertion attempts, ovarian cancer, previous CVC insertion, comorbidities and advanced disease being significant risk factors [1, 13].

In the present study we analyse our experience with PICC insertion in cancer patients and investigate the incidence of local and distant VTE and relative risk factors.

## Methods

This clinical audit consisted of a retrospective cohort study of all patients who received a PICC line between 1st January 2011 and 1st May 2014 at the Queens Centre for Oncology and Haematology, a tertiary cancer unit of the Hull and East Yorkshire NHS Hospitals Trust. All patients had active malignancy and required a PICC for chemotherapy or supportive treatment e.g. intravenous fluids or total parenteral nutrition. PICCs were inserted by a team of trained nurses under ultrasound guidance. The tip position was confirmed with chest radiograph, with the target site for the tip of the line being the cavo-atrial junction or the distal third of the superior vena cava.Lines were repositioned correspondingly. The PICC inserted is the Groshong® single lumen closed ended catheter under ultrasound guidance using the SonoSite Nanomaxx® machine. There was no routine use of thromboprophylaxis but if patients were taking antithrombotic therapy for other reasons this was recorded.

Patients developing PRDVT or distant VTE were all treated with therapeutic doses of LMWH as per institutional protocol. The agent used at our institute is Dalteparin and treatment was weight-adjusted, as per the SmPC for this agent.

The primary outcome of the analysis was the PRDVT. Data were collected for the secondary outcome of distant VTE (lower limb DVT and PE) at the same time. PRDVT was defined as symptomatic DVT in the upper limb vasculature ipsilateral to the PICC. Patient demographics, site and side of insertion, number of insertion attempts, catheter repositioning, reason for insertion, chemotherapy, cancer type, haemoglobin, white cell count and platelet count were prospectively recorded in a database for all patients undergoing a PICC insertion attempt. Outcome data was collected retrospectively by two of the authors (DJ, KW) who reviewed the electronic medical records of all patients including all radiology reports. All VTE-positive radiology reports documented the presence and site of thrombosis as per institutional guidelines. PRDVT and distant limb DVT was diagnosed with Doppler US in the presence of clinical suspicion (symptoms). PEs reported include both suspected (diagnosed with CTPA in the presence of symptoms) as well as incidentally-identified PEs (diagnosed with standard, 1 mm slice, CT thorax with intravenous contrast performed for cancer staging). Subsegmental PEs were included.

Time to PRDVT or distant VTE event was studied with the Kaplan Meier method and was calculated from the date of PICC insertion. Survival curves were compared using the log rank method. Logistic regression and Cox regression analyses were utilised to assess the effect of different baseline factors for the local (PRDVT), distant (lower limb DVT and PE) and combined (all VTE) endpoints. In all analyses a threshold of 5% was assumed for statistical significance.

## Results

During the study period 552 patients were referred for PICC insertion. Of these patients, 54 (9.8%) were excluded from the present analysis as they did not have a successful insertion. A further eight patients were excluded due to recording of incorrect patient ID numbers. This left 490 patients with complete data, eligible for analysis. Median follow-up was 286 days. The median age of patients was 65 with a range of 24 to 89. Two hundred seventeen patients were male. The most common primary diagnosis was colorectal cancer (*n* = 247, 50.4%), followed by breast cancer (*n* = 89, 18.2%) and pancreatic cancer (*n* = 39, 8%). PICC line was successfully inserted on the first attempt in 364 (74.3%) of patients. 42 (8.6%) patients were on antithrombotic therapy prior to PICC insertion [atrial fibrillation *n* = 6 (14%), previous VTE *n* = 32 (76%), thrombophilia *n* = 1, primary prophylaxis *n* = 1], the majority (*n* = 28, 67%) on LMWH (at therapeutic doses except one case on enoxaparin 40 mg o.d.). The most common reason for anticoagulation was a previous VTE (*n* = 26, 62%). Baseline groups and PRDVT/distant VTE incidence are shown in Table 1.

**Table 1:** Tabulation of patient demographics, baseline characteristics, PICC-related and distant DVT/VTE incidences

Characteristic	All patients (*n* = 490)	PICC-related DVT (*n* = 27)	Distant VTE (*n* = 26)
Age (Mean)	62.4	63.0	67.9
Gender			
Male	217 (44.3%)	13 (5.9%)	21 (9.7%)
Female	273 (55.7%)	14 (5.1%)	5 (1.8%)
Primary cancer			
Colorectal	247 (50.4%)	19 (7.6%)	22 (8.9%)
Breast	89 (18.2%)	6 (6.7%)	1 (1.1%)
Pancreas	39 (8%)	2 (5.1%)	1 (2.6%)
Other	115 (23.4%)	0	2 (1.7%)
Treatment intention			
Adjuvant	194 (39.6%)	16 (8.2%)	15 (7.7%)
Palliative	258 (52.7%)	11 (4.2%)	10 (3.9%)
Neo Adjuvant /Radical^a^	28 (5.5%)	0	0
Supportive^b^	10 (2.2%)	0	1 (10%)
Catheter insertion			
More than one insertion attempt	126 (25.7%)	11 (8.7%)	4 (3.1%)
Catheter repositioning	241 (49.2%)	15 (6.2%)	13 (5.4%)
Current anticoagulation	42 (8.6%)	3 (7.1%)	1 (2.4%)

*DVT* deep vein thrombosis, *PICC* peripherally inserted central catheter, *VTE* vascular thromboembolism

One patient had both PICC related DVT and distant VTE (PE)

^a^Neo adjuvant/Radical – Treatment with curative intent

^b^Supportive – intravenous fluids or total parenteral nutrition

PRDVT developed in 27 (5.5%) of the 490 patients in the cohort. Distant VTE developed in 26 patients (5.3%). All-type VTE (i.e. PRDVT or distant VTE) was identified in 52 (10.6%) patients with one patient having a PICC related DVT followed by a PE. Median time until the development of PICC-related DVT, other VTE and all type VTE was 21 days 95% Confidence Interval [CI] (14, 28), 55 days 95% CI (29, 81) and 43 days 95% (CI – 23, 63) respectively. The incidence of PE was 4% (*n* = 21) corresponding to 40% of all-type VTE events. The majority (*n* = 17, 81%) of PE events were incidental findings in staging CT thorax. One (5%) patient with PE had a concomitant subclavian and axillary vein PRDVT. Median time to PE was 55 days 96% CI (19, 91) amongst the patients who developed PRDVT the most proximal vein affected was the jugular in 4 (14.8%) patients, the subclavian in 14 (51.8%), the axillary in 4 (14.8%) and the brachial in 5 (18.5%).

Amongst the 42 patients on anticoagulation at the time of PICC insertion, 4 all-type VTE events were observed (9.5%) including 3 PICC-related DVT’s and one distant VTE at a median of 54 days 95% CI (0, 115).

Exploratory survival analyses indicated eight factors with potential predictive value. These factors were included in a logistic regression model (Table 2).

**Table 2:** Logistic regression model of factors with potential predictive value indicated by exploratory survival analyses

Variable	PICC related DVT		Distant VTE		All VTE	
	Odds Ratio (95%CI)	*p*	Odds Ratio (95%CI)	*p*	Odds Ratio (95%CI)	*p*
Male	0.97 (0.41,2.28)	0.94	3.82 (1.34,10.87)	0.01*	2.03 (1.04,3.93)	0.04*
Age (cont.)	1.01 (0.98,1.05)	0.54	0.97 (0.93,1.01)	0.24	0.99 (0.96,1.02)	0.53
More than one insertion attempt	2.61 (1.12,6.05)	0.03*	0.69 (0.22,2.16)	0.53	1.53 (0.76,3.06)	0.22
Catheter repositioning	1.29 (0.57,2.96)	0.54	1.44 (0.61,3.41)	0.41	1.25 (0.86,2.35)	0.47
Current anticoagulation	1.16 (0.31,4.39)	0.82	0.22 (0.02,1.74)	0.21	0.54 (0.17,1.68)	0.15
White Cell Count	1.10 (0.95,1.28)	0.19	1.14 (0.96,1.34)	0.13	1.12 (1.00,1.26)	0.04*
Fluropyrimidine containing chemotherapy	4.27 (1.30,14.07)	0.02*	7.77 (0.95,63.3)	0.06	4.54 (1.63,12.61)	0.01*
Bevacizumab containing chemotherapy	Not applicable	Not applicable	0.57 (0.12,2.65)	0.48	0.25 (0.06,1.08)	0.06

*DVT* deep vein thrombosis, *PICC* peripherally inserted central catheter, *VTE* vascular thromboembolism, *CI* confidence interval

* = *P* <0.05 statistically significant

Logistic regression (Table 2) indicated that requiring more than one attempt at insertion of PICC line more than doubled the risk of PICC related DVT (Odds Ratio [OR] 2.61, 95% Confidence Interval [CI] 1.12,6.05). The use of Fluoropyrimidine chemotherapy increased the risk by four-fold (OR 4.27, 95% CI 1.30, 14.07). No other factor was a significant predictor of PRDVT. When considering distant VTE events, male gender was a significant risk factor (OR 3.82 95% CI 1.34, 10.87) and when all VTEs were considered together, fluoropyrimidine containing chemotherapy (OR 4.54 95% CI 1.63, 12.61), being male (OR 2.03 95% CI 1.04, 3.93) and white cell count (OR 1.12 95% CI 1.00, 1.26) were significant.

## Discussion

PRDVT is a common problem for cancer patients, which could interrupt potential cancer treatment and cause significant morbidity. This study found a PICC related DVT rate of 5.5% which is in keeping with the rate found in the two other studies on PICC related DVT in cancer patients [1, 13].

A recent study which looked at PICC related thrombosis in all hospital patients found a slightly lower PICC related DVT rate of 3% in a population almost devoid of cancer patients [7]. The same study found a number of significant risk factors for developing a PICC related DVT which included previous DVT, PICC size, and surgery lasting >1 h [7]. Grove et al. [14] reported an overall venous thrombosis rate of 3.9%. This rate was different when the lines were inserted by nurses (4.5%) compared with radiologists (3.7%). They also identified that DVT risk correlated to catheter diameter, with DVT rates of 0, 1, 6.6 and 9.8% for catheters sized <3-F, 4-F, 5-F and 6-F respectively, comparable (albeit slightly higher) to the relative numbers reported by Evans et al. The importance of lumen size was further highlighted in a follow-on study by Evans et al. [15]. Cotogni and Pittiruti identified inappropriate choice of central venous access device and insertion technique as important risk factors for post-procedure complications, particularly in critically ill patients [16]. In our study the catheter used was uniform and all PICC insertions were conducted by nurses under ultrasound guidance.

In the study by Aw et al. on PICC line insertion in cancer patients, none of the above mentioned variables were predictive of PRDVT. However, the study reported that co-morbidities such as diabetes, COPD and advanced cancer did predict DVT [1]. Lee et al. identified previous catheterisation, more than one insertion attempt and ovarian cancer as being associated with an increased incidence of DVT [13].

This is the only study to date to specifically consider the rate of distant VTEs following PICC insertion in cancer patients. We found distant VTEs in 26 patients of which 20 had PEs and six had leg DVTs. One patient had a PICC related DVT and went on to develop a PE. Male gender was found to be a predictive factor. Whilst it is recognised that cancer itself is a significant risk factor for VTE, the published rate of VTE in cancer patients was 2% in a large study of 40 million cancer patients [17]. The rate of distant VTE is our study was 5.3% which suggests that PICC insertion might be considered as a possible risk factor for distant VTE as well.

Whilst LMWH is recommended as the current treatment for oncology patients who have suffered a VTE [18], anticoagulation did not exhibit a statistical effect on the development of local or distant VTE in our cohort. Current evidence suggests that thromboprophylaxis to prevent these events has not been shown to be effective, despite a number of randomised controlled trials (RCTs). For example, the two larger prospective studies, one of LMWH [19] and one of warfarin (low fixed and International Normalised Ratio [INR]-adjusted dosing) [20] did not achieve their primary endpoints. Moreover a recent Cochrane Database meta-analysis of 12 RCTs enrolling a total of 3611 patients assessing either primary prophylaxis dose heparins or low dose vitamin K antagonists in cancer patients with CVCs failed to demonstrate any benefit on the major endpoints studied [21]. Therefore, current guidance does not recommend thromboprophylaxis in the setting of a long-term CVC in cancer patients.

The finding of the present study that the number of insertion attempts is a risk factor for PRDVT points to the role of disrupted endothelium during the process of difficult cannulation [22]. This finding is supported by Lee et al. [13] who found more than two attempts at insertion was a significant predictor of PRDVT. Of interest is the correlation with white blood cell count of all VTE (one of the factors noted by Khorana et al.) which could reflect tumour burden-related effects. Leukocytosis was an independent predictor of VTE in an analysis of patients taking part in the REAL-2 study two arms of which were treated with infusional chemotherapy requiring central venous access devices [23]. ‘Inflammation’ has been well correlated with VTE in the literature but the particular role of the raised neutrophil numbers also suggest that this could be a clinical parameter that reflects the increasingly recognised role of neutrophil extracellular traps in the initiation of VTE [24].

Through this study, we are able to report an influence of the type of chemotherapy on the rate of PRDVT. Chemotherapy has been shown to increase the risk for VTE in general by more than two-fold [25]. In our study in which the majority of our patients were receiving systemic antineoplastic treatment, 5-fluorouracil specifically was associated with an increased risk of PICC-associated thrombosis and all type VTE whilst a trend for an increased risk for distant VTE was also observed. This effect could be explained by the well-known pro-thrombotic and endothelial effects of 5-fluorouracil [26]. We also observed a trend for increased distant VTE with bevacizumab. It should be noted that our study did not capture arterial events which may be more relevant for this agent.

This study was subject to some limitations. It did not include thrombotic events associated with long-term skin tunnelled catheters, cited as having a lower incidence compared to PICC-related VTE [27]. We were not able to report on the effect of the catheter to vein diameter ratio on the incidence of thrombotic events since it is not part of the standard insertion protocol in our centre and therefore was not recorded. Preclinical evidence suggests that increasing catheter to vein diameter ratio may dramatically restrict blood flow through the veins [28, 29] whilst clinical data suggest that a catheter to vein diameter ratio of >45% may increase the likelihood of VTE 13-fold [30, 31]. As a retrospective cohort study, we were unable to obtain data on certain patient demographics such as body mass index, smoking status, comorbidities and patient mediations. This meant that we could not test the risk factors found by Aw et al., [1]. It is possible that the confounding variables we have been unable to record may have had an impact on PICC related DVT rates in this study. Selection bias was minimised by including all patients who had a successful PICC insertion in the study period. This study addressed suspected PRDVT events only and therefore did not capture subclinical thrombotic events that may have been identified with planned investigations. Conversely, this rendered the study within pragmatic confines, documenting clinically significant events.

Finally, it is of note that our institutional guidelines are compatible with the recent Michigan Appropriateness Guide for Intravenous Catheters (MAGIC) comprehensive recommendations, which aim to minimise PICC-related complications [29, 32]; however, the present study was conducted prior to the relevant publication and therefore did not investigate all corresponding care and maintenance end-points in individual patients.

## Conclusion

This is a large study looking at PRDVT, solely in cancer patients and confirms the incidence of PRDVT as documented in the literature. It also suggests that PICC insertion increases the risk of ‘distant VTE’ (PEs and lower limb DVTs) in these patients. This correlates with the findings of other studies of VTE’s associated with peripherally inserted central catheters in patients with, or without cancer [31, 32]. This study did not find any strong predictors of PICC-related DVT. To improve our understanding of DVT development associated with PICC insertion and the predicted risk factors, further large, prospective studies with carefully pre-planned data capture would be required.

## Abbreviations

CT: Computed Tomography (scan)
CTPA: CT pulmonary angiogramm
CVC: Central venous catheter
DVT: Deep vein thrombosis
INR: International Normalised Ratio
LMWH: Low Molecular Weight Heparin
OR: Odds Ratio
PE: Pulmonary embolism
PICC: Peripherally inserted central catheter
PRDVT: Peripherally inserted central catheter-related deep vein thrombosis
RCT: Randomised Control Trial(s)
US: Ultrasound
VTE: Vascular thromboembolism
